# Tear Film and Keratitis in Space: Fluid Dynamics and Nanomedicine Strategies for Ocular Protection in Microgravity

**DOI:** 10.3390/pharmaceutics17070847

**Published:** 2025-06-28

**Authors:** Ryung Lee, Rahul Kumar, Jainam Shah, Joshua Ong, Ethan Waisberg, Alireza Tavakkoli

**Affiliations:** 1 The Houston Methodist Research Institute, Houston, TX 77030, USA; 2 Department of Biochemistry and Molecular Biology, University of Miami Miller School of Medicine, Miami, FL 33136, USA; rahulca13@gmail.com; 3 Albert Einstein College of Medicine, Bronx, NY 10461, USA; jainam.shah@einsteinmed.edu; 4 Department of Ophthalmology and Visual Sciences, University of Michigan Kellogg Eye Center, Ann Arbor, MI 48105, USA; ongjo@med.umich.edu; 5 Department of Clinical Neurosciences, University of Cambridge, Cambridge CB2 1TN, UK; ethanwaisberg@gmail.com; 6 Department of Computer Science and Engineering, University of Reno, Reno, NV 89512, USA; tavakkol@unr.edu

**Keywords:** spaceflight, dry eye syndrome, keratitis, nanomedicine, tear film, ophthalmic solutions

## Abstract

Spaceflight-associated dry eye syndrome (SADES) has been reported among astronauts during both International Space Station (ISS) and Space Transportation System (STS) missions. As future missions extend beyond low Earth orbit, the physiological challenges of spaceflight include microgravity, radiation, and environmental stressors, which may further exacerbate the development of ocular surface disease. A deeper understanding of the underlying pathophysiology, along with the exploration of innovative countermeasures, is critical. In this review, we examine nanomedicine as a promising countermeasure for managing ophthalmic conditions in space, with the goal of enhancing visual health and mission readiness for long-duration exploration-class missions.

## 1. Introduction

The ocular surface is a fragile interface; the multi-layered tear film affects visual acuity and comfort. Composed of lipid, aqueous, and mucin layers, the tear film is continuously regulated through blinking, drainage, and evaporation. A single stimulus may result in altered function through feedback mechanisms and reflex loops [1] For instance, cytokines such as IL-10, IL-17A, and IFNγ are highly correlated with each other and associated with some DED signs [2] Under Earth’s gravity, these systems are well-characterized and vital for ocular surface health.

In microgravity, however, these homeostatic systems face significant disruption. The absence of gravity alters the basic physics of astronaut physiology. As a result, astronauts experience cephalad fluid shifts, cardiac deconditioning, loss of musculoskeletal mass, and changes in circadian rhythm [3,4]. Ocular physiology is not exempt; spaceflight has been associated with optic disk edema, globe flattening, and refractive changes, collectively described under spaceflight-associated neuro–ocular syndrome (SANS) [5]. Additionally, microgravity disrupts normal fluid mechanics on the ocular surface, potentially impairing tear film formation, distribution, and clearance [6,7]. These changes occur in parallel with space-induced immune dysregulation and oxidative stress, which may further compromise ocular health [8,9].

However, there is a paucity of literature on anterior segment conditions during spaceflight. This review aims to synthesize current knowledge on tear film dynamics in microgravity, with a focus on the underlying physical forces that govern tear behavior in space, especially the shift in dominance from gravity to surface tension and capillarity. We also explore how these altered dynamics contribute to dry eye syndrome, impaired tear clearance, and increased susceptibility to ocular surface disease during spaceflight. Finally, we highlight emerging strategies to mitigate these risks, including nanomedicine-based drug delivery systems designed to optimize ocular surface hydration, reduce inflammation, and restore tear film stability in the unique environment of microgravity. These insights may inform future approaches to preserving visual performance and ocular health during long-duration missions.

## 2. Fluid Dynamics in Spaceflight

In Earth’s gravity, fluid dynamics are governed by a balance of inertial, viscous, and gravitational forces. We can model hemodynamics using fluid dynamic equations [10] In microgravity, gravitational forces become negligible, and capillary and viscous forces dominate [11] This shift is reflected in several governing equations:

### 2.1. Navier–Stokes in Microgravity

In the low Reynolds number environment of tear film dynamics, inertial forces become negligible, and flow behavior is governed by the Stokes flow limit of the Navier–Stokes equations [12]:

ρ(∂v/∂t + (v · ∇)v) = −∇p + μ∇^2^v + f_surfacetension_

where

- v: velocity field of the tear film
- ρ: density of the tears
- μ: dynamic viscosity
- ∇p: pressure gradient (reduced in microgravity)
- f_surfacetension_: capillary force at fluid interfaces

In a microgravity setting, where bulk motion is minimal, the inertial terms diminish, simplifying the equation to:

0 = −∇p + μ∇^2^v + f_capillary_

This reflects a Stokes flow regime where surface tension plays a dominant role [13]

### 2.2. Capillary Number

The Capillary Number (Ca) quantifies the ratio of viscous to surface tension forces. The capillary number for tear film deposition is typically 1.9 × 10^−3^ [14,15]:

Ca = μU/γ

where

- μ: dynamic viscosity
- U: characteristic velocity (e.g., blink-induced tear motion)
- γ: surface tension

In microgravity several changes are postulated to occur: surface tension remains constant, but characteristic velocity may decrease due to the absence of gravity-induced drainage. This results in the capillary number decreasing because of dominant surface tension forces. This can impair tear spreading and drainage, leading to abnormal ocular surface wetting [16]

### 2.3. Bond Number

The Bond Number (Bo) evaluates the relative importance of gravitational to surface tension forces:

Bo = Δρ g L^2^/γ

where

- Δρ: density difference between tears and air
- g: gravitational acceleration (approaches zero in space)
- L: characteristic length scale (e.g., tear meniscus height)
- γ: surface tension

In microgravity:


g → 0 ⇒ Bo → 0

This confirms that gravity’s influence on tear dynamics becomes negligible, and surface tension becomes the primary force governing tear film behavior. Surface tension completely dominates, leading to tear pooling and lack of drainage [17] These principles predict a fundamental reorganization of tear film dynamics, including stagnation, pooling, and impaired drainage [18] The changes in fluid dynamics in spaceflight lead to uneven tears forming dome-shaped pools on the eye surface, incomplete redistribution during blinking, altered lipid spreading, impaired corneal wound healing, and impaired tear clearance, leading to and potentiating SADES [6,19]

### 2.4. Immune Dysregulation and Risk of Infection in Microgravity

**Figure 1:**
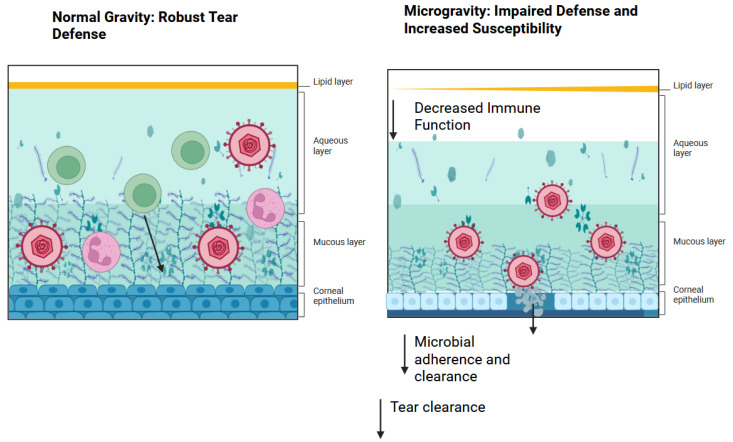
Pathogenesis of viral keratosis in spaceflight. As a result of decreased tear film secretions, immunity on the ocular surface becomes impaired, leading to decreased microbial adherence and clearance. Created in BioRender. Lee, R. (2025) https://BioRender.com/r61p8im.

Perturbations of the immune system are thought to arise in the context of spaceflight. Elevations in the plasma concentrations of TNF, IL-1α, and IL-1β have been observed among astronauts [20] These key inflammatory cytokines are typically released during early immune responses and essential in the clearance of many pathogens [20] Furthermore, in the period immediately after spaceflight, experimental records have demonstrated diminished natural killer cell function, altered peripheral leukocyte distribution, diminished monocyte function, diminished granulocyte function, and dysregulated T cell intracellular signaling [21] Clinical symptoms associated with immune dysregulation were reported affecting 46% of crew members including the reactivation of herpetic viruses [22,23] For viral keratitis, the immune system plays a crucial role, including both natural killer T cells and cytotoxic T cells [24] The modulation of cytokines, namely IL-6, is known to reduce the damage seen in corneas with fungal keratitis [24] Furthermore, inflammation may mediate and cause variability in drug response and toxicity by altering the regulation of drug-metabolizing enzymes and transporters [25] Along with changes in the tear film, immune system dysregulation may increase the risk of complications from infectious keratitis during exploration class missions (Figure 1).

### 2.5. Impact on Mission

**Figure 2:**
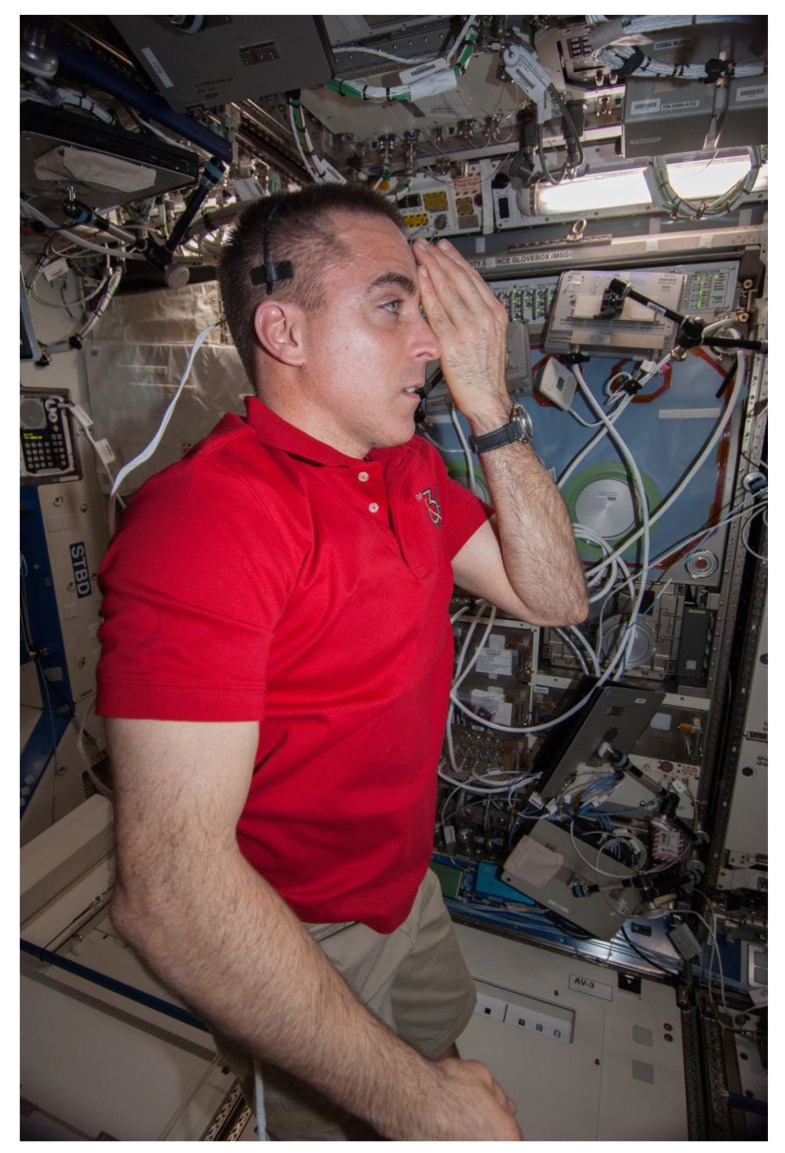
NASA astronaut Chris Cassidy, Expedition 36 flight engineer, performs a visual exam using an eye chart (out of frame) in the Destiny laboratory of the International Space Station. Vision is crucial to the success of the astronaut mission. Image courtesy of NASA.

Given the changes in the tear film during microgravity, tears will accumulate on the ocular surface, forming large, dome-shaped pools. The thicker tear film may distort the refractive surface of the cornea, leading to blurry and fluctuating vision [26] Visual integrity is critical to the operational success of space missions, where tasks often demand high visual acuity, rapid focus adjustment, and consistent ocular comfort in dynamic environments (Figure 2) [27] Its impact goes beyond ocular discomfort, as DED has been consistently linked to reduced contrast sensitivity, increased light scatter, and higher-order aberrations, all of which compromise visual performance. These disturbances can manifest as blurring, glares, or halos, potentially interfering with navigation, manual docking, and digital equipment interpretation onboard spacecraft [28]

## 3. Keratitis in Spaceflight

a. Viral Keratitis

The reactivation of latent herpesviruses, including the varicella zoster virus (VZV) and Epstein–Barr virus, has been consistently observed in astronauts during and after spaceflight. This phenomenon is hypothesized to be driven by spaceflight-induced immune dysregulation, characterized by increased levels of stress hormones (cortisol, catecholamines), a Th1 to Th2 immune shift, and reduced cytotoxic T cell and NK cell function, which are essential for controlling latent viral infections [20,29]. Though astronauts are generally asymptomatic, the presence of VZV DNA and infectious VZV particles in saliva during and after spaceflight has been well-documented. In a study by Cohrs et al. [30], infectious VZV was cultured from the saliva of two astronauts within 2 days of landing following a 13-day mission, despite the absence of clinical zoster [30]. HSV-1 DNA has also been intermittently detected, and anti-HSV-1 antibody titers have been shown to rise during spaceflight, further supporting subclinical reactivation [23,30] These findings suggest that astronauts can shed infectious viruses without clinical signs, a phenomenon with potential implications for ocular health [30]

Viral keratitis, a corneal inflammation caused by the reactivation of HSV or VZV in the trigeminal ganglion, remains a theoretical but significant concern in the spaceflight environment. While no documented cases of ocular herpes reactivation (e.g., herpes simplex keratitis or herpes zoster ophthalmicus) have occurred during flight to date, the detection of VZV and HSV-1 in astronaut saliva and the known neurotropic reactivation pathways of these viruses raise concerns for reactivation in the ophthalmic branch of the trigeminal nerve [31] Stress, radiation exposure, and sleep deprivation, common in microgravity missions, are all established triggers of ocular viral reactivation on Earth [32] Should ocular reactivation occur in space, the consequences could be severe, including corneal scarring or opacity, decreased visual acuity, endothelial damage or uveitis, and difficulties in diagnosis and management without a slit-lamp biomicroscope [33,34] Furthermore, treatment options aboard spacecrafts may be limited. While antivirals like acyclovir or valacyclovir could theoretically be stored in mission medical kits, their effectiveness depends on early recognition, which may be compromised by limited ophthalmologic equipment and crew training.

The risk of viral keratitis may be amplified on long-duration missions, where cumulative immune suppression, prolonged confinement, and radiation exposure intersect. Notably, reactivation rates for VZV and CMV increase significantly during International Space Station (ISS) missions compared to shorter shuttle flights, and VZV has been found in up to 65% of astronauts on long missions [35] These findings emphasize the need for pre-flight vaccinations, real-time salivary monitoring, and the development of point-of-care viral diagnostics for ocular pathogens.

b. Bacterial Keratitis

Bacterial keratitis, an infection of the cornea that can lead to rapid vision loss if untreated, presents a particularly concerning risk during long-duration spaceflight. On Earth, bacterial keratitis is commonly caused by pathogens such as *Pseudomonas aeruginosa*, *Staphylococcus aureus*, and *Streptococcus pneumoniae*, especially in contact lens wearers or individuals with ocular surface compromise [36,37] In space, the risk is heightened due to a confluence of host-related, microbial, and environmental factors unique to the space environment [38]

Spaceflight induces a well-documented suppression of both innate and adaptive immunity. Microgravity, psychological stress, radiation exposure, and disrupted circadian rhythms contribute to alterations in immune regulation, particularly in the attenuation of pathways such as NF-κB and reduced antigen presentation [20] This dysregulation compromises the host’s ability to fight infections and has been associated with the reactivation of latent viruses and potentially opportunistic bacterial pathogens. Increased cortisol and stress-related immunosuppression may predispose astronauts to ocular infections, including bacterial keratitis [39]

Spacecrafts such as the ISS represent closed, semi-sterile ecosystems where microbial contamination arises from astronauts’ skin, GI flora, delivered supplies, and onboard equipment. Despite rigorous sterilization protocols, studies have identified viable microbial communities including *Pseudomonas* spp., *Klebsiella pneumoniae*, *Staphylococcus* spp., and even multidrug-resistant organisms [40] Many of these bacteria have been isolated from spacecraft surfaces and air and are capable of forming biofilm-structured microbial communities resistant to disinfection and antibiotics [41] Biofilm formation has been documented on the ISS, with potential for colonization on ocular surfaces, especially under compromised tear film conditions.

Several experiments have demonstrated that microgravity enhances bacterial virulence and adaptability. For instance, *Pseudomonas aeruginosa*, a leading cause of contact lens-related keratitis, shows increased biofilm formation, stress resistance, and growth rate under simulated microgravity. This may lead to altered morphologies, which maintain effects in microgravity [42] Similarly, *Salmonella enterica* becomes more virulent in mice under modeled microgravity, while *E. coli* and *S. aureus* exhibit altered antibiotic susceptibility profiles in flight conditions [43] These changes are partly driven by the upregulation of global stress regulators such as the RNA binding protein, Hfq, and quorum-sensing systems, which enhance pathogenicity in confined environments [44]

There are historical precedents for serious infections in space. During the Apollo 13 mission, one astronaut developed a *Pseudomonas* infection, raising early concerns about bacterial adaptation in space [45] While keratitis was not explicitly documented, the causative organism is a known ocular pathogen. On the ISS and Mir, various bacterial genera, including Staphylococcus epidermidis and Propionibacterium acnes, were found on multiple surfaces [46] These commensals can become pathogenic under immunocompromised conditions, potentially affecting ocular structures like the cornea. A decline in beneficial bacteria, such as *Faecalibacterium prausnitzii* and *Lactobacillus* spp., correlates with increased inflammatory susceptibility and reduced epithelial defense, further heightening the risk of keratitis [47]

In summary, bacterial keratitis represents a credible threat during spaceflight due to immune suppression, microbial adaptation, and compromised ocular defense. Future missions must prioritize infection prevention protocols, real-time microbial diagnostics, and robust pharmacological strategies to ensure ocular health and crew safety.

c. Fungal Keratitis

Fungal keratitis is a vision-threatening ocular infection typically caused by filamentous fungi, such as *Aspergillus*, and *Fusarium,*, or by yeast-like fungi, such as *Candida*. In terrestrial settings, risk factors include ocular trauma, contact lens use, and immunosuppression [48] Although fungal keratitis remains rare in spaceflight, emerging data on microbial persistence and astronaut-associated fungal colonization raises concerns regarding the potential for opportunistic ocular infections during long-duration missions [49] Several studies have demonstrated the presence of fungal biota aboard the International Space Station (ISS), including *Aspergillus*, *Penicillium*, *Cryptococcus*, and *Rhodotorula* species [50] These organisms are commonly found in terrestrial environments but are capable of opportunistic infection, particularly in immunocompromised individuals. HEPA filtration and controlled humidity aboard the ISS mitigate airborne spread; however, fungal spores are lightweight and remain suspended in microgravity, posing potential inhalation and mucosal exposure risks [51] Longitudinal studies of astronaut microbiota have confirmed the transient colonization of *Candida albicans* and other fungi in nasal, pharyngeal, and salivary samples, although in-flight fungal isolation rates were significantly lower compared to pre- and postflight samples [52] Notably, *C. albicans* was detected in saliva using onboard loop-mediated isothermal amplification assays, emphasizing the feasibility of in situ fungal monitoring.

Environmental isolates such as *Rhodotorula mucilaginosa* have been recovered from crew members in-flight, suggesting potential colonization or secondary contamination from environmental surfaces [53]. Despite the reduced diversity and viability of cultivable fungi in space likely due to unfavorable growth conditions such as limited nutrients, low humidity, and controlled air quality fungi capable of biofilm formation or possessing melanin-based protective adaptations may persist [54] In fact, melanized fungi such as *Cryptococcus neoformans* have demonstrated increased viability under spaceflight conditions compared to their non-melanized counterparts [54] Melanin appears to confer resistance to oxidative and radiation-induced stress, supporting its role in fungal survival in space environments [55]

The implications for ocular health are particularly relevant for long-duration missions. *Candida albicans*, for instance, is a known cause of endogenous fungal endophthalmitis and keratitis, particularly in hosts with disrupted epithelial barriers or altered immunity [56] Given that astronauts experience immune modulation, including impaired antigen presentation, reduced NK cell function, and increased stress hormones, there exists a theoretical vulnerability to fungal infections, including keratitis, especially in the context of corneal micro-abrasions or contact lens wear [57] Table 1 compares the reactivation of infectious keratitis on Earth to those in spaceflight.

Furthermore, spaceflight-associated dry eye syndrome may exacerbate the risk of fungal keratitis by compromising the protective tear film and ocular surface defenses. The reduction in blink-induced meibum distribution and decreased tear clearance in microgravity may create a microenvironment more susceptible to colonization [58] Though no documented cases of fungal keratitis have been reported in spaceflight to date, the presence of viable opportunistic fungi, immune suppression, and ocular surface alterations warrant continued surveillance.

## 4. Nanomedicine

In recent years, nanotechnology has emerged as a promising platform for targeted ocular drug delivery in spaceflight, where conventional formulations are limited by rapid tear clearance and altered ocular surface dynamics. Various nanocarriers including liposomes, chitosan-based nanoparticles, dendrimers, and polymeric micelles have been engineered to enhance drug retention, penetration, and sustained release on the ocular surface [59,60,61] While this is a field of ongoing research, cutting-edge methods for the synthesis of organic nanoparticles include: supramolecular self-assembled aggregates, polymeric nanoparticles, DNA-polymer conjugates, and dendrimers [62] Liposomes, for example, have a tendency to self-assemble, allowing for control and modification of their core composition [63]. These chitosan nanoparticles, for instance, are mucoadhesive and can increase pre-corneal residence time, whereas dendrimers offer precise control over size and surface functionalization, enabling multivalent drug binding [63,64] Methods such as ionic gelation (for chitosan NPs), solvent evaporation (for liposomes), and nanoprecipitation (for polymeric micelles) allow for the fine-tuning of drug release profiles and high relative encapsulation efficiencies [65,66] Early approaches in ocular nanomedicine with liposomes and dendrimers, focused on overcoming tear drainage and improving corneal permeability; however, more recent innovations include contact lens embedded nanoparticles, carbon-based nanostructures, and inorganic nanostructures that allow sustained and minimally invasive delivery, critical advantages for long-duration missions [67,68,69] While comparative studies in space analogs remain limited, these systems hold significant potential for improving keratitis prophylaxis and treatment in microgravity.

a. Dry Eye Syndrome

Spaceflight-associated dry eye syndrome (SADES) represents a unique clinical challenge due to the altered physiology and environmental stressors encountered in microgravity. Astronauts experience changes in tear film stability, meibomian gland dysfunction, increased tear evaporation, and reduced blinking frequency, compounded by environmental stressors such as low humidity, decreased air pressures, extravehicular activities, high CO_2_ levels, and radiation exposure [70] These factors collectively exacerbate ocular surface inflammation and tear film instability, predisposing astronauts to DED during and after missions [71]

Conventional topical therapies, such as artificial tears or corticosteroid drops, are impractical in microgravity due to fluid handling limitations and suboptimal pharmacokinetics. The low ocular surface residence time and potential for bottle misapplication pose logistical and clinical hurdles [72] Additionally, many medication eye drops have limited shelf lives. During exploration class missions, such as the minimum 21-month trip to Mars, current formulations will not suffice. Furthermore, space-induced immune dysregulation and oxidative stress can reduce the efficacy of standard anti-inflammatory agents [73]

Nanomedicine presents a promising countermeasure tailored to the unique constraints of spaceflight. Advanced nanoformulations, such as nanoemulsions, liposomes, dendrimers, chitosan, and nanostructured lipid carriers (NLCs), may enhance drug bioavailability, improve ocular surface adhesion, and offer controlled-release capabilities [74,75]

Notably, FDA-approved nanoemulsions like Restasis and Cequa deliver cyclosporine A in stable nanosized particles, significantly improving ocular tissue penetration and tear film retention, features potentially advantageous in low-gravity environments where dosing frequency must be minimized [65] Recent innovations such as hyaluronic acid-coated liposomes, ROS-scavenging cerium oxide nanoparticles, and thermoresponsive hydrogels loaded with corticosteroids or FK506 have demonstrated efficacy in mitigating inflammation and oxidative damage in terrestrial DED models [76]

These platforms are particularly relevant to SADES, where prolonged oxidative stress and inflammation may result from both microgravity-induced immune dysregulation and radiation exposure. Moreover, contact lens-based delivery systems incorporating drug-loaded nanoparticles offer an elegant solution to zero-gravity drug retention challenges [77] These lenses can continuously release therapeutic agents over days, reduce the need for manual instillation, and protect the ocular surface from microdebris and dehydration [78]

Despite the promise, the clinical translation of nano-based therapies to space remains limited. Key challenges include ensuring temperature stability over long-duration missions, biocompatibility with altered astronaut physiology, and rigorous in-flight delivery validation. As NASA and international space agencies plan for extended lunar and Martian exploration, the deployment of nanoformulated ocular therapeutics may become essential in safeguarding astronaut ocular health.

b. Keratitis

Keratitis poses a significant risk to astronaut ocular health, particularly under spaceflight conditions that compromise tear film integrity, alter immune responses, and elevate oxidative stress. In microgravity, impaired lymphatic drainage, increased exposure to radiation, and changes in stress hormone levels collectively attenuate the immune system and potentiate microbial colonization on the ocular surface [31] Fungal keratitis, in particular, is difficult to treat with conventional topical therapies due to poor corneal penetration and rapid drug clearance. Nanomedicine offers a transformative solution for space-based ocular infections. ROS-responsive nanocarriers such as the nanocarrier, GC-EB-VOR, formulated with glycol chitosan and 4-carboxyphenylboronic acid pinacol ester, enable targeted, sustained release of antifungal agents like voriconazole at the site of infection [79] ROS-reactive formulations, such as GC-EB, are highly effective in spaceflight where there is a higher prevalence of reactive oxygen species [80] These nanodrugs enhance corneal retention, scavenge ROS, and minimize inflammatory damage, effectively overcoming spaceflight-induced pharmacokinetic challenges [80] Future application of such stimuli-responsive nanosystems may enable compact, long-duration ophthalmic treatment strategies aboard spacecrafts, significantly improving astronaut ocular safety and preserving vision in extended missions.

c. Spaceflight Associated Neuro–Ocular Syndrome

Spaceflight-associated neuro–ocular syndrome (SANS) is a constellation of neuro-ophthalmic changes increasingly recognized among astronauts on long-duration missions. These changes include optic disk edema, globe flattening, choroidal folds, and hyperopic shift, attributed in part to cephalad fluid redistribution in microgravity and subsequent alterations in cerebrospinal fluid (CSF) dynamics [81] Elevated intracranial pressure (ICP) relative to intraocular pressure (IOP), along with impaired venous outflow and glymphatic drainage, is hypothesized to play a central role in the pathophysiology of SANS [82] However, the syndrome remains incompletely understood, and effective pharmacological interventions have not been established [83]

Acetazolamide, a carbonic anhydrase inhibitor traditionally used for glaucoma and idiopathic intracranial hypertension, has been proposed as a potential countermeasure for SANS due to its dual action in reducing both IOP and CSF production. Acetazolamide has been applied previously to astronauts with elevated ICP, with mixed results. However, systemic administration of acetazolamide in astronauts poses logistical and physiological challenges, including fluid electrolyte imbalance, renal load, and undesirable sulfonamide-related side effects are particularly concerning in austere space environments where medical interventions are limited [84]

In this context, nanotechnology-enabled localized delivery of acetazolamide may offer a transformative approach to mitigate SANS. The encapsulation of acetazolamide in elastin-like recombinamer (ELR)-based nanocarriers via the supercritical antisolvent (SAS) technique offers a paradigm shift in ocular pharmacotherapy suited for spaceflight conditions [85] These ELR-based nanoparticles, measuring around 42 nm and exhibiting long-term colloidal stability (zeta potential −33 mV), can traverse ocular surface barriers and allow for sustained drug release, thereby avoiding systemic toxicity while maintaining pharmacologic efficacy [85] The biocompatibility and thermoresponsive behavior of ELRs further enable their performance in dynamic environments such as microgravity, where ocular surface hydration, tear film stability, and fluid mechanics are altered [86]

In-flight administration of acetazolamide-loaded ELRs may achieve targeted reduction in IOP without the risks of systemic administration. Moreover, combining this approach with intravitreal or subconjunctival implants using materials such as PLGA or ELR hydrogels could provide long-acting drug reservoirs, which is critical for mission durations extending beyond six months. These delivery systems may also help fine-tune the translaminar pressure gradient between IOP and ICP, an emerging therapeutic target in the management of SANS [87] As spaceflight increasingly ventures toward extended lunar and Martian habitation, integrating nanotechnological drug delivery platforms such as ELR-based acetazolamide carriers holds great promise. They offer precise pharmacokinetics, enhanced ocular bioavailability, and reduced toxicity, aligning with the demands of personalized aerospace medicine. Future studies should evaluate the pharmacodynamics of these systems under simulated microgravity and validate their efficacy in both preventing and reversing SANS-related neuro–ocular changes.

## 5. Conclusions

Given the high likelihood of tear film disruption and ocular surface instability during spaceflight, we have outlined the key biophysical mechanisms underlying these changes in microgravity. This review outlines the complications that may arise from altered tear dynamics, such as impaired vision, increased susceptibility to infection, and inflammation, and explores emerging nanomedicine-based countermeasures. We also propose that future studies evaluate the integration of these advanced therapeutics into NASA’s existing medical kits to address spaceflight associated conditions such as SANS (spaceflight-associated neuro–ocular syndrome) and SADES (spaceflight-associated dry eye syndrome). With these innovations, we may be better equipped to preserve ocular health and visual performance during long-duration exploration-class missions. Future clinical validation and the integration of nanopharmaceuticals into astronaut medical kits may become indispensable for next-generation planetary missions.

## Figures and Tables

**Table 1:** Comparison of microbial keratitis on Earth versus Spaceflight.

Feature	Bacterial Keratitis (Earth)	Bacterial Keratitis (Spaceflight)	Viral Keratitis (Earth)	Viral Keratitis (Spaceflight)	Fungal Keratitis (Earth)	Fungal Keratitis (Spaceflight)
Immune Response	Rapid innate and neutrophilic response	Impaired immunity, reduced neutrophil function	Cell-mediated immunity controls HSV	T cell suppression may prolong/reactivate virus	Granulomatous inflammation	Impaired macrophage function
Tear Film Defense	Normal antimicrobial peptides, lysozyme	Reduced tear turnover, compromised defense peptides	Adequate tear-based viral inhibition	Decreased mucin, lysozyme activity	Tear defenses resist fungal invasion	Compromised tear barrier
Pathogen Virulence	Well-characterized strains; localized infections	Potential increase in virulence (space radiation, stress)	Herpesviruses show latency/reactivation patterns	Reactivation is more likely due to immune dysregulation	Geographic fungi cause trauma-related infection	Unknown pathogenicity in space environment
Ocular Surface Integrity	Intact epithelial renewal	Microgravity reduces epithelial repair rate	Reactivation often at corneal periphery	Healing delayed in microgravity	Epithelial barrier maintains resistance	Barrier integrity reduced
Clinical Presentation Risk	Predictable based on exposure	Risk increases due to unpredictable microbe behavior	Controlled unless immunocompromised	Higher likelihood of recurrence	Rare, trauma-induced	Possibly more severe or persistent

## Abbreviations

The following abbreviations are used in this manuscript:

SADES	Spaceflight-Associated Dry Eye Syndrome
SANS	Spaceflight-Associated Neuro–ocular Syndrome
IFNγ	Interferon Gamma
IL-10	Interleukin-10
IL-17A	Interleukin-17A
NK cells	Natural Killer Cells
NF-κB	Nuclear Factor Kappa-Light-Chain-Enhancer of Activated B Cells
ROS	Reactive Oxygen Species
NLCs	Nanostructured Lipid Carriers
ELR	Elastin-Like Recombinamer
SAS	Supercritical Antisolvent (technique)
PLGA	Poly(lactic-*co*-glycolic acid)
